# Kinetic Behavior of Quaternary Ammonium Hydroxides in Mixed Methane and Carbon Dioxide Hydrates

**DOI:** 10.3390/molecules26020275

**Published:** 2021-01-07

**Authors:** Muhammad Saad Khan, Cornelius Borecho Bavoh, Khor Siak Foo, Azmi Mohd Shariff, Zamzila Kassim, Nurzatil Aqmar Bt Othman, Bhajan Lal, Iqbal Ahmed, Mohammad Azizur Rahman, Sina Rezaei Gomari

**Affiliations:** 1 Mechanical Engineering Department, Texas AM University at Qatar, Al Rayyan 5270, Qatar; 2 Chemical Engineering Department, Universiti Teknologi PETRONAS, Darul Ridzuan 32610, Perak, Malaysia; bavohcornelius@gmail.com (C.B.B.); khorsiakfoo88@gmail.com (K.S.F.); azmish@utp.edu.my (A.M.S.); 3 CO_2_ Research Centre (CO_2_RES), Universiti Teknologi PETRONAS, Seri Iskandar 32610, Perak, Malaysia; 4 PETRONAS Research Sdn Bhd, Kawasan Institusi Bangi, Lot 3288 3289 Off Jalan Ayer Itam, Kajang 43000, Selangor, Malaysia; zamzila_kassim@petronas.com (Z.K.); nurzatil.othman@petronas.com (N.A.B.O.); 5 Mechanical Engineering Department, King Abdulaziz University of Science and Technology, Jeddah 80200, Saudi Arabia; irajboot@kau.edu.sa; 6 Petroleum Engineering Department, Texas AM University at Qatar, Al Rayyan 5270, Qatar; marahman@tamu.edu; 7 School of Computing, Engineering and Digital Technologies, Teesside University, Middlesbrough TS1 3BX, UK; s.rezaei-gomari@tees.ac.uk

**Keywords:** kinetic hydrate inhibition, ammonium hydroxides, formation rate, induction time, mixed gas hydrates

## Abstract

This study evaluates the kinetic hydrate inhibition (KHI) performance of four quaternary ammonium hydroxides (QAH) on mixed CH_4_ + CO_2_ hydrate systems. The studied QAHs are; tetraethylammonium hydroxide (TEAOH), tetrabutylammonium hydroxide (TBAOH), tetramethylammonium hydroxide (TMAOH), and tetrapropylammonium hydroxide (TPrAOH). The test was performed in a high-pressure hydrate reactor at temperatures of 274.0 K and 277.0 K, and a concentration of 1 wt.% using the isochoric cooling method. The kinetics results suggest that all the QAHs potentially delayed mixed CH_4_ + CO_2_ hydrates formation due to their steric hindrance abilities. The presence of QAHs reduced hydrate formation risk than the conventional hydrate inhibitor, PVP, at higher subcooling conditions. The findings indicate that increasing QAHs alkyl chain lengths increase their kinetic hydrate inhibition efficacies due to better surface adsorption abilities. QAHs with longer chain lengths have lesser amounts of solute particles to prevent hydrate formation. The outcomes of this study contribute significantly to current efforts to control gas hydrate formation in offshore petroleum pipelines.

## 1. Introduction

For over a century, the global increase in energy demand, due to economic progress, has predominantly been satisfied by hydrocarbon-based fossil fuels. Crude oil, natural gas, and coal, now provide over 80% of the primary energy supply worldwide [1]. Among the stated fossil fuels, natural gas is considered the most abundant and more eco-friendly and is known to emit fewer amounts of greenhouse gases [2].

The distribution of natural gas reserves differs geographically according to the physical locations worldwide. Natural gas reservoirs enriched with carbon dioxide (CO_2_) can be found in several parts of the world, especially in the South-East Asian region. Countries such as Indonesia, Malaysia, and Thailand, are renowned for their high-carbon natural gas reserves [3,4]. In Malaysia, gas reserves of over 70 and 87 moles per cent CO_2_ respectively exist in the K5, and J7 fields situated offshore of Sarawak [5,6]. The CO_2_ content of natural gas not only reduces its energy content (calorific value) but also increases its refining costs as well [7,8]. Also, the presence of CO_2_ in natural gas causes various issues, such as severe problems of flow assurance and pipeline integrity due to gas hydrate formation and corrosion, especially in deep-water wells [7,9].

Gas hydrates are crystalline solids in which gas molecules are shielded and stabilized by Van der Waal’s forces in hydrogen-stricken water molecules [3,10,11,12,13,14,15]. The formation of gas hydrates is the main flow-assurance challenge in the offshore oil and gas sector, contributing to severe blockages during the process of hydrocarbon output to pipeline transport and refining facilities in all fields [16,17]. To minimize such losses, a new discipline known as flow assurance engineering has emerged [18,19,20]. Flow assurance becomes more significant as oil and gas exploration and field development progress into deeper water (500 m), where longer pipelines in hostile operating environments are prone to gas hydrate formation [21]. Deepwater production faces various technical challenges, such as, (1) lower operating temperatures inside hydrate formation regions and (2) inconsistent production profiles due to factors such as gas composition, pressure, temperature, and oil and liquid content over the lifespan of the field [19,22,23].

Techniques such as water exclusion, chemical suppression, heat circulation and depressurization, are the main methods widely used to prevent gas hydrate deposits in offshore pipelines [24,25,]. Nonetheless, in most offshore situations, the most feasible solution for deepwater gas pipelines, is the chemical suppression technique, mainly because of its practicability and cost-efficiency [26,27]. Thermodynamic hydrocarbon inhibitors (THIs) and low-dose hydrate inhibitors (LDHIs) are the chemical hydrate suppression methods available. THIs are organic solvents that can create a connection between hydrogen and water, thus, lowering the liquid-vapor hydrate equilibrium (HLVE) temperature. Meanwhile, LDHIs include two types: kinetic hydrate inhibitors (KHIs); typically consisting of surfactants, which are water-soluble polymers and, anti-agglomerates (AAs) [28,29,30]. KHIs unlike THIs, do not alter hydrate nucleation structures and their phase boundary conditions. In comparison, AAs in general, do not inhibit the formation of hydrate, but rather, they create a transmittable slurry that prohibits the aggregation of hydrate crystals from forming superior plug-in structures that obstruct pipelines [31].

Water-soluble polymers such as polyvinyl pyrrolidone (PVP) and polyvinyl caprolactam (PVCap), are the most widely used KHIs. Karaaslan et al. [32] claimed that PVP and polyoxyethylene (PEO) are KHIs; however, they contain cancerous materials that have significant human health and safety implications when utilized. Also, PEO’s influence on hydrate mitigation is relatively less than PVP [32].

The petroleum industry generally uses both THIs and KHIs with different chemical constituents in varying amounts, which leads to high costs of operation when used in larger quantities. These limitations have drawn much attention from both industry and researchers to find efficient compounds that could provide dual-functional hydrate inhibition impact. Quaternary ammonium salts are green compounds with excellent thermal stability in nature, which are tailor-made for task-specific applications and especially for gas hydrate inhibition. Li et al. [33] first introduced quaternary ammonium compounds salts as gas hydrate inhibitors. They reported that tetramethylammonium chloride (TMACl) could inhibit hydrates better than imidazolium-based ionic liquids. Another study by Tariq et al. [34] confirmed that quaternary ammonium compounds exhibit both thermodynamic and kinetic hydrate inhibitory potentials.

Recently, Khan and co-workers have worked extensively on the evaluation of quaternary ammonium compounds as thermodynamic hydrate inhibitors for CH_4_, CO_2_, and their mixed gas hydrates [4,6,8,9,11,25,27,35,36,37,38,39,40,41,42]. Their results show that quaternary ammonium hydroxide (TMAOH) best inhibits hydrate formation by reducing the hydrate dissociation temperature by 2.1 K [6]. Although the thermodynamic hydrate inhibition of quaternary ammonium compounds has been studied extensively, their KHI behavior is still not fully understood. Also, the effect of quaternary ammonium compounds on high CO_2_ content natural gas system is not well understood, thus, requiring investigations that could be useful to manage hydrate formation challenges in high CO_2_ natural gas production fields and around the globe [4,6,35,42].

Therefore, this present study investigated the kinetic hydrate inhibition influence of quaternary ammonium hydroxides on three (3) different CH_4_ + CO_2_ mixed gas systems. The KHI measurements taken include the total gas consumed, induction time, and initial rate of hydrate formation in the binary mixed gas-hydrate systems of 30% CH_4_ + 70% CO_2_, 50% CH_4_ + 50% CO_2_, and 70% CH_4_ + 30% CO_2_ using the isochoric constant cooling method. Experiments were carried out at simulated pipeline pressures between 3.50 to 7.50 MPa at temperatures of 274.0 and 277.0 K. The test was performed at two different experimental temperatures to allow the evaluation of the subcooling effect. The subcooling temperature is the difference between the hydrate equilibrium temperature (*T*_eq_) and the experimental temperature (*T*_ex_) as described in methodology section later. The kinetic inhibition results of the QAHs were also compared to a commercial kinetic hydrate inhibitor (PVP) to confirm their potentials as adequate replacements for the conventional gas hydrate inhibitors in the industry.

## 2. Results and Discussion

### 2.1. Influence of QAHs on Induction Time of Mixed Gas Hydrates

**Figure 1:**
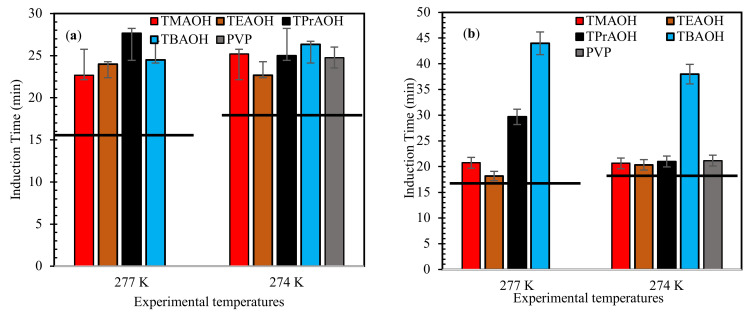

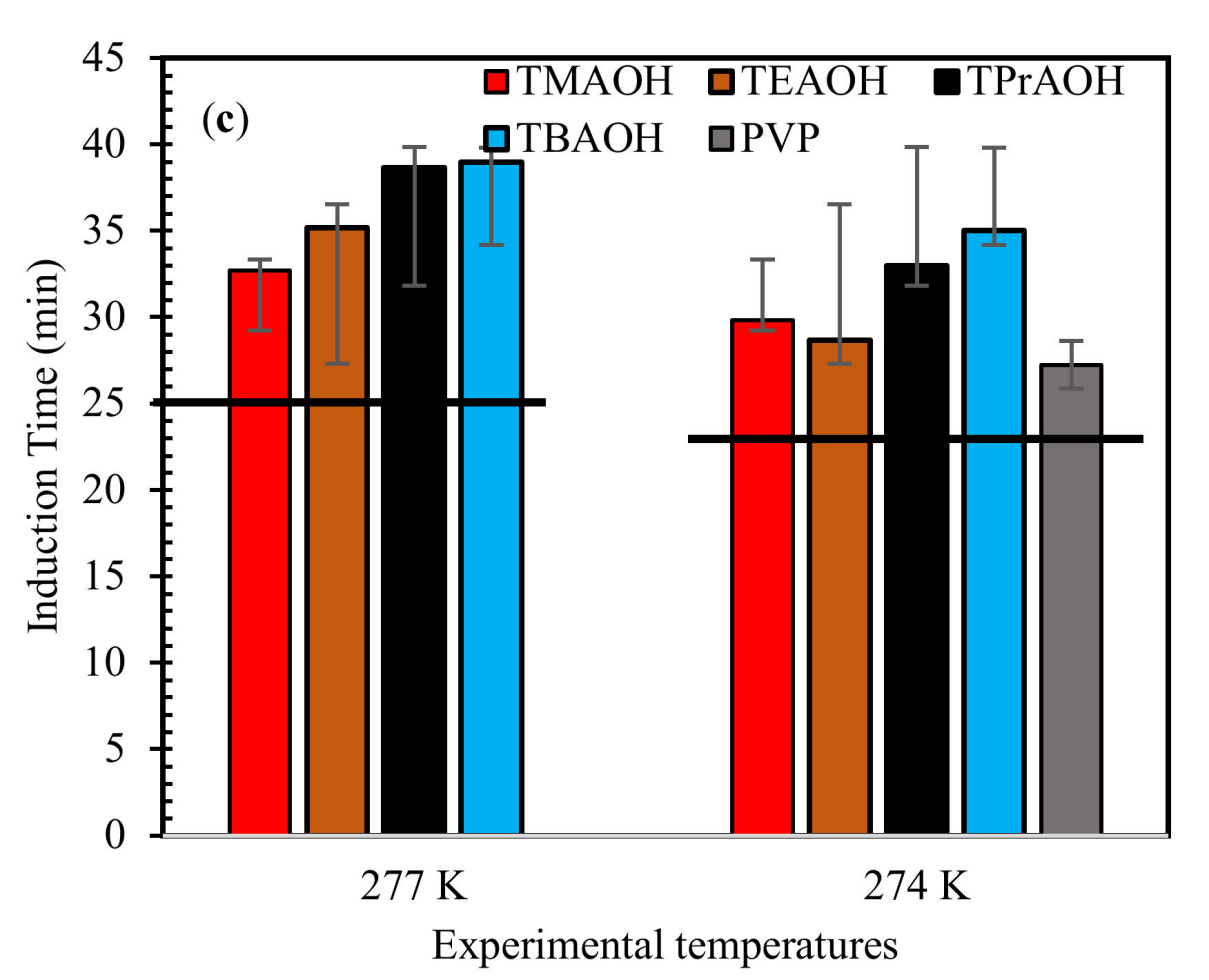
Influence of 1 wt% QAHs on induction times of mixed gas hydrates at different experimental temperatures for (**a**) 70% CH_4_ + 30% CO_2_, (**b**) 50% CH_4_ + 50% CO_2_, and (**c**) 70% CH_4_ + 30% CO_2_, and comparison with the commercial inhibitor (PVP). The solid lines represent pure water.

Figure 1 presents the measured induction time results for the tested QAHs + mixed gas hydrate systems under the studied experimental conditions. The findings for the QAHs tested are also compared with PVP tested at 274.0 K experimental temperature. From Figure 1, the induction time for all the QAH systems examined can be seen to increase compared with the pure water system. The induction time for the lower subcooling conditions (277.0 K) was less than that for, the higher subcooling temperature (274.0 K). A potential reason for this observed behavior is the presence of a higher subcooling difference (Δ*T* 9 K), which enhances the metastable (hydrate forming) region, resulting in shorter induction times. This behavior was also reported in earlier studies, which indicate that sufficient subcooling temperature is required for efficient nucleation [43,44]. Also, in a subcooling temperature system, the hydrate nucleation and formation behavior has been proven to be controlled by the activation barrier of the system [45]. Thus, at higher subcooling temperature there is a large negative entropy of activation which causes the hydrate to form faster and grow more compared to systems at lower subcooling [45]. The induction time results suggest that all of the QAHs considered were able to work as kinetic inhibitors.

The kinetic hydrate inhibition strength of the studied QAHs increased by increasing their alkyl chains. QAHs with chain lengths above two (TPrAOH and TBAOH) exhibited the best hydrate inhibition impact due to their increased hydrophobic activity arising from their cation functionality. The increased hydrophobic nature of TPrAOH and TBAOH causes an improved barrier between the gas-liquid interfaces [46,47], reducing the dissolution of gas into the bulk liquid phase. On the other hand, the ability of QAHs to delay hydrate formation is similar to PVP. This confirms the weakness of PVP at high subcooling conditions [48]. Therefore, using QAHs as high subcooling conditions could provide similar hydrate nucleation delays as PVP.

### 2.2. Influence of QAHs on Relative Inhibition Performance of Mixed Gas Hydrates

**Figure 2:**
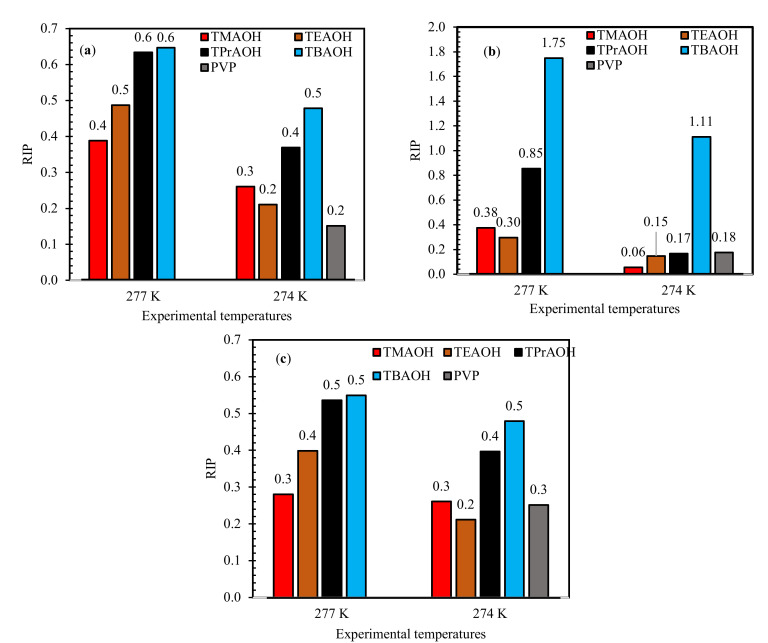
Influence of 1 wt% QAHs on relative inhibition power (RIP) of mixed gas hydrates at different experimental temperatures for (**a**) 70% CH_4_ + 30% CO_2_, (**b**) 50% CH_4_ + 50% CO_2_, and (**c**) 70% CH_4_ + 30% CO_2_, and comparison with commercial inhibitor (PVP).

For a better understanding of the inhibition strength of the QAHs, the estimated RIP*_induction time_* values at both temperatures are presented in Figure 2. In Figure 2, the RIP values of the mixed gas + QAHs systems exhibited relatively less inhibition strength compared with pure CH_4_ and CO_2_ hydrates in literature [3]. This behavior is observed perhaps due to the presence of relatively higher subcooling temperature differences in the mixed gas conditions tested in the work. For instance, the subcooling of pure systems [3] was found between 7.0–9.0 K, whereas in mixed gas cases, it varied between 9.50–10.50 K. In comparison to those of the pure gas hydrates CH_4_ and CO_2_, the values of RIP*_induction time_* for the QAHs considered indicate that they offer comparable inhibition to that of the commercial inhibitor PVP and show their ability to worked in relatively higher subcooling conditions. The RIP*_induction time_* data also show the influence of subcooling. At the higher subcooling temperature of 274.0 K, the RIP*_induction times_* values of the QAHs are observed to be higher than at 277.0 K. This is due to the large negative entropies of activation generated at, the higher temperature [45].

### 2.3. Influence of QAHs on Initial Formation Rate of Mixed Gas Hydrates

**Figure 3:**
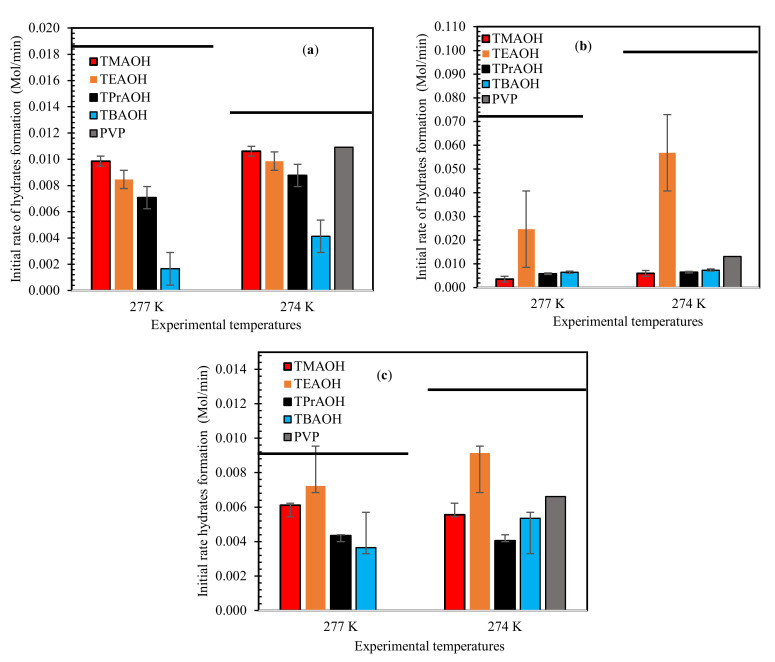
Impact of 1 wt% of QAHs on initial mixed gas hydrate formation at different experimental temperatures for (**a**) 70% CH_4_ + 30% CO_2_, (**b**) 50% CH_4_ + 50% CO_2_, and (**c**) 70% CH_4_ + 30% CO_2_, and comparison with the commercial inhibitor PVP. The solid lines represent pure water.

The initial rate of hydrate formation in 1 wt% QAH-mixed gas systems at test temperatures of 277.0 K and 274.0 K are recorded in Figure 3. The influence of subcooling temperature was evident, as the initial rate of hydrate formation was high at 274.0 K compared to 277.0 K. The rate of hydrate formation was inhibited with increasing QAHs chain length. In the hydrate nucleation and growth process, the amount of gas disbanded in the liquids stage can help. Often, the rate of gas dissolution as a result of mass transfer depends on the surface tension of the liquid phase [49,50,51]. The surface tension of QAHs decreases with increasing alkyl chain, according to Kartikawati et al. [52], which inhibits the rate of gas dissolution to form more hydrates. This better kinetic inhibition impact occurs because of the increasing free energy at the gas-liquid interfaces in the presence of QAHs with longer chain lengths. In the case of TBAOH [52], there is an increase in the surface adsorption to the hydrate nucleus crystals, which aids in providing a better inhibition effect.

The gas chromatography (GC) analysis performed for all the mixed gas samples was basically to test which guest molecules are highly consumed during hydrate formation. It was observed that the amount of CO_2_ in the gas mixtures was significantly decreased compared to its initial composition before hydrate formation. This means that CO_2_ hydrates are formed more than CH_4_.

### 2.4. Influence of QAHs on Consumption of Mixed Gas Hydrates

**Figure 4:**
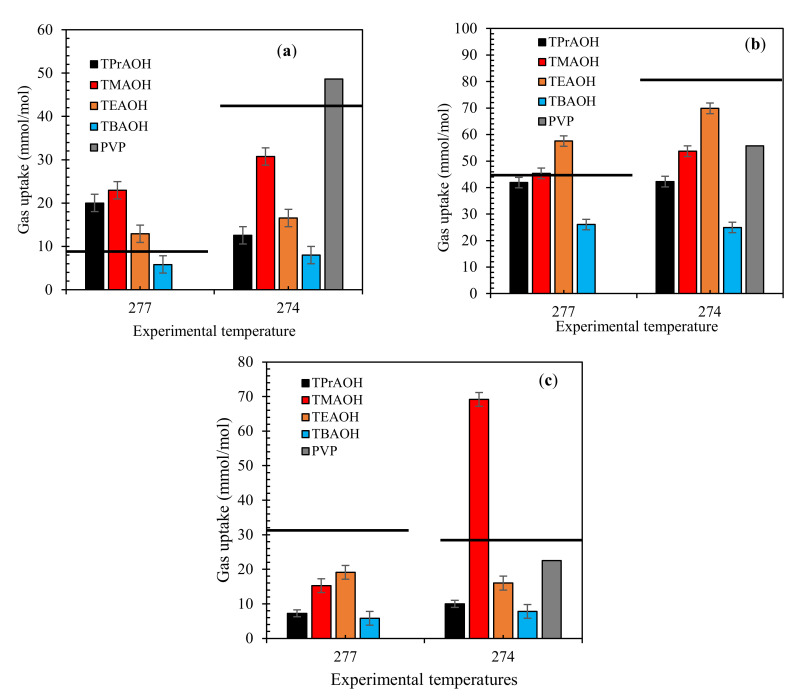
Effect of 1 wt% QAHs on gas uptake by mixed gas systems at different temperatures for (**a**) 70% CH_4_ + 30% CO_2_, (**b**) 50% CH_4_ + 50% CO_2_, and (**c**) 70% CH_4_ + 30% CO_2_, and comparison with the industrial inhibitor PVP. The solid lines represent pure water.

The amount of gas uptake in the presence or absence of QAH systems is shown in Figure 4. The results for the mixed gas systems show that the QAHs were able to decrease the amount of gas consumed into hydrates. Increasing the QAHs alkyl chain length further decreases the mixed gas uptake (moles consumed). This can be attributed to its surface-active nature of the QAHs, this causes the QAHs to adequately adhere to the gas-liquid interface, causing hydrate inhibition. This confirms the performance of TBAOH solutions as the best QAH in all tested systems.

As reported previously, the studied QAHs possess strong THI influence for CH_4_, CO_2_, and different binary mixed gas systems. This study discusses the kinetic impact of QAHs on binary mixed CH_4_ + CO_2_ gas hydrates. The results in this work and form our previous studies [5,53,54] show that these QAHs works effectively as dual-functional gas hydrate inhibitors. Their THI impact decreases with increasing alkyl chain because of poor hydrogen bonding affinity, thus TMAOH exhibits the best thermodynamic inhibition impact [53]. On the other hand, the kinetic hydrate inhibition effects of QAHs is increased with alkyl chain length due to their higher hydrophobicity. For this reason, QAHs should have optimal alkyl chain lengths, such as with ethyl (C_2_H_5_) or propyl (C_3_H_8_) molecules. This will achieve greater dual functionality, providing adequate hydrogen bonding while increasing surface adsorption at the gas-liquid interface. QAHs can provide sufficient steric obstacles in various ways, leading to the delayed nucleation of hydrates. We further recommend that the effect of other quaternary ammonium compounds (QAC) and ionic liquids should be tested for binary gas mixture for better data comparison and understanding.

### 2.5. QAHs Molar Concentration Effect on Hydrate Formation

The understanding of the molar concentration effect of the studied QAHs on their hydrate inhibition impact would provide significant insights for their applicability. Considering the concentration limit (2 wt%) for KHIs application in the industry, additives with excellent hydrate inhibition effect at less molar concentration are mostly desired. This is because, there would be less amount of the additive in the produced water or natural gas system, thus, making gas processing and produce water treatment easy and free from complex complications. The molar concentration of QAHs is controlled by their molar masses (Table 1). The equivalent molar concentration of the studied QAHs at 1 wt% are; TMAOH (0.002 mol%), TEAOH (0.0012 mol%), TPAOH (0.0009 mol%), and TBAOH (0.0007 mol%), suggesting that the amount of moles of the QAHs reduces in the solution with increasing QAHs chain length. Based on the findings in the work, the hydrate inhibition effect of the QAHs is enhanced with increasing chain length (in wt%). Interestingly, the QAHs with longer chain length inhibits hydrate formation with less molar concentration. This finding affirms that the best performing QAHs KHIs (TPAOH and TBAOH) can mitigate hydrate formation with less amount of solute particles in the system. If the TMAOH molar concentration is normalized to 1, then the relative molar concentration of TEAOH is 0.62, TPAOH is 0.45 and TBAOH is 0.35. This implies that 0.35 moles of TBAOH can mitigate hydrate formation more than 1 mole of TMAOH. Thus, QAHs with longer chain lengths have less amount of solute particles to prevent hydrate formation. However, further investigations are needed to fully understand the impact of QAHs equivalent molar concentrations on hydrates nucleation and crystallization.

## 3. Materials and Methods

### 3.1. Materials and Sample Preparation

The chemicals employed in this study are described in Table 1. All of the compounds tested were used without further purification. The required concentrations of the QAHs in all the samples were prepared with deionized water. An HR-250AZ analytical balance with a precision of ±0.1 mg (AD Company, Japan) was used for the accurate weight measurements of specimens. Several reported hydrate-based kinetics studies are measured in mol% [55,56]. However, the equivalent concentration in mol% and wt% of inhibitors exhibit a significant difference in their inhibition trends which could affect their inhibition impact analyses using either concentration unit [57,58]. In most cases, both concentration units result in an opposing inhibition impact or trend of discussion. However, in most industrial applications, wt% is preferable and widely used. Since this work is focused on industrial applications, using wt% was the appropriate concentration unit to provide relevant results interpretation that will contribute more towards practical field testing of QAHs.

### 3.2. Experimental Set-Up and Kinetic Measurement

A stainless-steel high-pressure cell reactor was used to run all the kinetic hydrate tests in this work.

**Figure 5:**
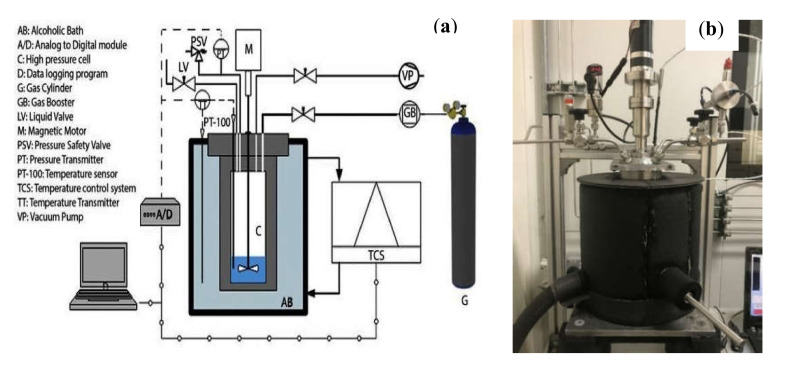
Experimental set-up used in this study: (**a**) schematic; (**b**) Image of the actual setup.

The test apparatus consists of a high-pressure cell of 650 mL in volume, which operates effectively between 253–523 K, with a maximum operating pressure of 20 MPa. Further details of the experimental set-up and the operating procedure can be found elsewhere [11,42,59,60,61]. Figure 5 shows the schematic and the actual experimental set-up used in this study.

### 3.3. Kinetic Measurements of Gas Hydrate Inhibitors

**Figure 6:**
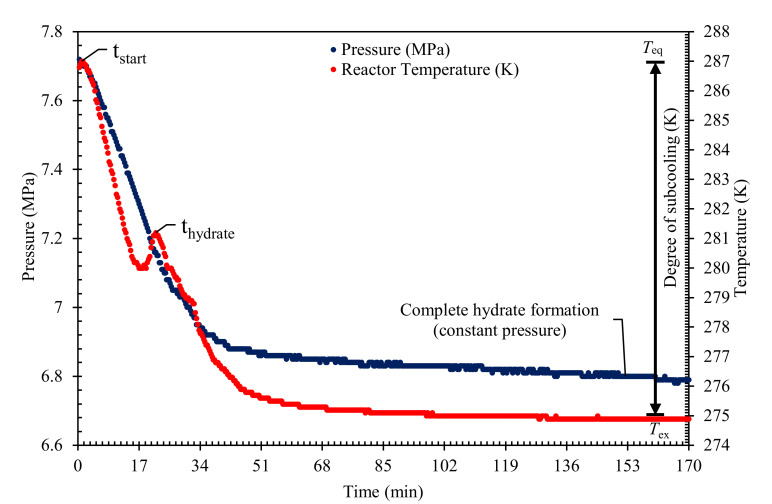
A typical Time versus pressure and temperature plot to indicate the induction time and gas uptake during hydrate formation in a constant cooling method.

An isochoric constant cooling system was used in all the kinetic testing assessment. All the QAHs solutions were prepared with deionized water for a 1 wt% solution of each QAH and PVP (commercial KHI). The mixed gas systems used in this work are 30% CO_2_ + 70% CH_4_, 50% CO_2_ + 50% CH_4_, and 70% CO_2_ + 30% CH_4_. Table 2 summarizes the experimental conditions used in this study. The KHI performance was evaluated based on the retardation of nucleation (induction time), inhibition of crystal growth rate, or total gas uptake. The complete details of the kinetics measurement and KHI evaluation procedures adopted can be found in our earlier publications [4,5,42,54]. To conduct the experiments, the cell was cleaned to remove contaminants, then 100 mL of the desired QAHs solution was loaded into the cell. The cell was then immersed in the water bath and vacuum. The required mixed gas system was injected into the cell to the required experimental pressure. Then the system was left to stabilize for about 3 h at the initial testing conditions. The hydrate formation test was initiated by reducing the system temperature to the experimental temperature shown in Table 2. The stirrer was switched on at 400 rpm and the data logging program begun simultaneously to initiate the experiment. The hydrate formation was confirmed by observing a sharp increase in the system temperature and a simultaneous decrease in the pressure as described in Figure 6. When constant pressure is attained in the hydrate cell (for about five hours), the testing was terminated and considered complete.

#### 3.3.1. Induction Time Measurement

The induction time (*t_induction_*) describes the inhibitor’s ability to delay hydrate nucleation process before visible hydrate growth occurs [62,63]. It is the time required to form a detectable hydrate phase volume [64,65,66]. Nevertheless, the induction period is often defined as a probabilistic phenomenon because of the non-stochiometric existence of hydrate formation. Therefore, both tests have been replicated at least twice, and the mean values were reported.

The induction time in this study is calculated according to the isothermal processes as reported in the literature [5,42,53] and from Figure 6 as shown in Equation (1):

(1) $$t_{\mathrm{induction}}=t_{\mathrm{hydrate}}-t_{\mathrm{start}}$$

where *t_induction_* refers to the time taken for hydrate nucleation to occur, *t_start_* is the system’s initial condition prior to the beginning of the experiments, and *t*_hydrate_ is the point at which measurable hydrate were observed, evident by a rapid decrease in pressure as shown in Figure 6. The significant rise in the temperature peak further indicates the formation of hydrates owing to the exothermic nature of hydrate formation. In Figure 6, the initial pressure decreases between *t_start_* and *t_hydrate_* show the induction time ‘*t_induction_*’ before gas consumes at the nucleation of gas hydrate, this defines the area of catastrophic hydrate formation (see Figure 6).

#### 3.3.2. Relative Inhibition Performance

The relative inhibition performance technique proposed by Koh et al. [67] was used to effectively compared the hydrate inhibition impact of the QAHs. The method was used to account for the kinetic system-dependency effect of the tested inhibitors. The relative inhibition performance (RIP*_induction time_*) factor was estimated using Equation (2). RIP*_induction time_* values 0 correspond to a superior hydrate inhibitory performance:

(2) $${\mathrm{RIP}}_{\mathrm{induction}}={\frac{\mathrm{induction}\;{\mathrm{time}}_{\mathrm{inhibitor}}-\mathrm{induction}\;{\mathrm{time}}_{\mathrm{water}}}{\mathrm{induction}\;{\mathrm{time}}_{\mathrm{water}}}}_{n}$$

where *n* is the number of QAHs tested in this work.

#### 3.3.3. Gas Chromatography (GC) Analysis of Mixed Gas Hydrates

Owing to the guest cage density with each product, the final gas composition of the mixed gas system was observed to differ from the initial composition during the gas hydration process. To solve this issue, a gas chromatography (Perkin Elmer Clarus 580, Shelton, CT, USA) was used to determine the amount of gas remaining in the reactor after hydrates were completely formed. The gas chromatography findings are used to measure the amount in moles of the mixed gas present (referred to as ‘*f*’ in Equation (3) below). Also, in the presence and absence of QAHs, the gas chromatography values indicate the composition of mixed gas hydrates consumed during the hydrate formation process.

#### 3.3.4. Total Mixed Gas Uptake

During the hydrate formation cycle, the level of gas absorbed during hydrate formation can be determined using the real gas equation, which estimates the difference between the number of moles of gas as shown in Equation (3) [68,69,70]:

(3) ΔnH=VRPzTf−PzT0

where *V* is the system gas phase volume, R is the universal gas constant, *P* and *T* is the system pressure, and Temperature, respectively, *z* is the compression factor calculated from the Peng-Robinson state equation [71,72]. The subscripts *0* and *f* are the amounts of moles of gas at time zero and complete hydrate formation, respectively.

#### 3.3.5. Initial Formation Rate

The initial formation rate of hydrate shows precisely how rapidly it is formed. By finding the plot gradient of measured moles of initial methane consumed during the hydrate formation process versus the time elapsing before hydrate formation, as described by Partoon et al. [73], the initial hydrate formation rate is determined Equation (4) as follows:

(4) $$\frac{dn}{dt}=k_{gas}\left(n_{H_{f}}-n_{H}{}_{o}\right)$$

where *k_gas_* is the hydrate formation rate constant, *n_Hf_* are the total moles of gas uptake at any time *f*, and *n_Ho_* is the moles of gas uptake at time zero.

## 4. Conclusions

In the present work, the kinetic behavior of QAHs has been evaluated for different binary CH_4_ + CO_2_ mixed gas hydrate systems at percentage proportions of 70:30, 50:50, and 30:70 of CH_4_ + CO_2_. Kinetically, all the studied QAHs inhibit the formation of mixed gas hydrates by increasing the hydrate formation induction time and decreasing the initial rates of hydrate formation. However, the reduction in gas uptake during hydrate formation is evident in all the systems studied. The trend of kinetic inhibition is found to depend on the type of gas system involved. Due to the different interactions between QAHs molecules and the gas molecules. The hydrate inhibition is more efficient with high CO_2_ hydrates compared to CH_4_ hydrates systems. However, the QAHs with longer alkyl chains (TPrAOH and TBAOH) gave better inhibition than those with shorter chains (TMAOH and TEAOH). TPrAOH and TBAOH exhibited superior kinetic inhibition performance overall for all systems, which is comparable to the commercial inhibitor PVP. All the QAHs systems studied reduced the initial hydrate formation rates more than PVP at 274 K, especially at 1 wt%. On the other hand, PVP performed reasonably well compared to the QAHs with shorter alkyl chains (TMAOH and TEAOH) in delaying hydrate formation. Slowing down hydrate nucleation and growth by disrupting the activity of water and gas dissolution via adsorption, together with lowering the subcooling temperature, are the possible mechanisms for the kinetic hydrate inhibition observed in all the studied systems. Therefore, applying these efficient dual-functional gas hydrate inhibitors in offshore transmission pipelines could provide a viable solution to the problems associated with gas hydrate formation in the industry.

## Figures and Tables

**Table 1:** Details of chemicals employed.

Chemical	Purity (wt%)	MW (g mol^−1^)	Formula	Supplier
Water	Deionized	18.02	H_2_O	Self-prepared
Mixed gas (30% CO_2_ + 70% CH_4_)	99.00%	24.43	-	Gas Walker Sdn Bhd
Mixed gas (50% CO_2_ + 50% CH_4_)	99.00%	30.02	-	Gas Walker Sdn Bhd
Mixed gas (70% CO_2_ + 30% CH_4_)	99.00%	35.62	-	Gas Walker Sdn Bhd
Tetrabutylammonium hydroxide	99.00%	259.47	TBAOH	Merck Millipore
Tetraethylammonium hydroxide	99.00%	147.26	TEAOH	Merck Millipore
Tetrapropylammonium hydroxide	99.00%	203.36	TPrAOH	Merck Millipore
Tetramethylammonium hydroxide	99.00%	91.15	TMAOH	Merck Millipore
Polyvinylpyrrolidone	99.00%	160,000	PVP	Merck Millipore

**Table 2:** Details of KHI experimental conditions encountered in gas transmission lines for various gases with or without aqueous QAHs solutions.

Mixed Gas Composition	Temperature (K)	Pressure Range (MPa)
30% CO_2_ + 70% CH_4_	274.0 and 277.0	7.50
50% CO_2_ + 50% CH_4_	274.0 and 277.0	6.50
70% CO_2_ + 30% CH_4_	274.0 and 277.0	5.0

## Data Availability

The data will be available on request.

## Sample Availability

Not available.

## Nomenclature

Abbreviation	Description
QACs	Quaternary Ammonium Compounds
QAHs	Quaternary Ammonium Hydroxides
CO_2_	Carbon dioxide
CH_4_	Methane
AAs	Anti-agglomerates
GC	Gas chromatography
HLVE	Hydrate liquid vapor equilibrium
KHIs	Kinetic hydrate inhibitors
LDHIs	Low-dosage hydrate inhibitors
Δ*n_H_*	Gas uptake in hydrate phase (moles)
PEO	Polyethylene oxide
PVCap	Polyvinyl caprolactum
PVP	Polyvinyl pyroledinium
RIP_induction_	Relative inhibition performance
TMACl	Tetramethylammonium chloride
THIs	Thermodynamic hydrate inhibitor
TMAOH	Tetramethylammonium hydroxide
TPrAOH	Tetrapropylammonium hydroxide
TBAOH	Tetrabutylammonium hydroxide
*t_induction_*	Induction time (min)
TEAOH	Tetraethylammonium hydroxide
*V*	Gas-phase volume
*P*	Pressure
R	Gas constant
*z*	Gas compressibility factor
*T*	Temperature
C_2_H_5_	Ethane
C_3_H_8_	Propane
ΔT	Subcooling temperature
subscripts	
*f*	Final
*k*	Rate constant
*0*	Time zero
*H*	Hydrates
*Units*	
K	Kelvin
MPa	Megapascal
wt%	Wight percent
mmol/mol	Millimoles of gas per moles of water
min	Minute
Mol/min	Moles per minute
mL	Milliliters
rpm	Revolutions per minute
h	Hours
